# Obesity, Insulin Resistance, Caries, and Periodontitis: Syndemic Framework

**DOI:** 10.3390/nu15163512

**Published:** 2023-08-09

**Authors:** Lorena Lúcia Costa Ladeira, Gustavo Giacomelli Nascimento, Fábio Renato Manzolli Leite, Silas Alves-Costa, Janaína Maiana Abreu Barbosa, Claudia Maria Coelho Alves, Erika Barbara Abreu Fonseca Thomaz, Rosangela Fernandes Lucena Batista, Cecilia Claudia Costa Ribeiro

**Affiliations:** 1 Postgraduate Program of Dentistry, Federal University of Maranhão, São Luís 65085-580, MA, Brazil; lorena.lucia@discente.ufma.br (L.L.C.L.); silas.alves@discente.ufma.br (S.A.-C.); cmcoelhoa@gmail.com (C.M.C.A.); ebthomaz@gmail.com (E.B.A.F.T.); 2 National Dental Research Institute Singapore, National Dental Centre Singapore, Singapore 168938, Singapore; ggn@dent.au.dk (G.G.N.); fabio@duke-nus.edu.sg (F.R.M.L.); 3 Oral Health Academic Clinical Programme (ORH ACP), Duke-NUS Medical School, Singapore 169857, Singapore; 4 Section for Periodontology, Department of Dentistry and Oral Health, Aarhus University, 8000 Aarhus, Denmark; 5 Postgraduate Program of Public Health, Federal University of Maranhão, São Luís 65020-070, MA, Brazil; janaina.bs@discente.ufma.br (J.M.A.B.); rosangela.flb@ufma.br (R.F.L.B.)

**Keywords:** syndemic, insulin resistance, obesity, caries, periodontitis, adolescents

## Abstract

(1) Background: To investigate the grouping of obesity and insulin resistance with caries and periodontitis from a syndemic perspective through pathways of socioeconomic inequalities, smoking, alcohol, and high sugar consumption in adolescence. (2) Methods: The population-based RPS Cohort study, São Luís, Brazil, in ages 18–19 years (*n* = 2515) was used. The outcomes were the grouping of pbesity and Insulin Resistance Phenotype (latent variable formed by Triglycerides/HDL ratio, TyG index, and VLDL) and the Chronic Oral Disease Burden (latent variable comprising caries, bleeding on probing, probing depth ≥ 4 mm, clinical attachment level ≥ 3 mm, and visible plaque index ≥ 15%). Socioeconomic Inequalities influencing the Behavioral Risk Factors (latent variable formed by added sugar, smoking, and alcohol) were analyzed using structural equation modeling. (3) Results: Socioeconomic Inequalities were associated with the Chronic Oral Disease Burden [Standardized Coefficient (SC) = 0.222, *p* < 0.001]. Behavioral Risk Factors were associated with increased Chronic Oral Disease Burden (SC = 0.103; *p* = 0.013). Obesity was associated with the Insulin Resistance Phenotype (SC = 0.072; *p* < 0.001) and the Chronic Oral Disease Burden (SC = 0.066; *p* = 0.005). The Insulin Resistance Phenotype and the Chronic Oral Disease Burden were associated (SC = 0.053; *p* = 0.032). (4) Conclusion: The grouping of obesity and early events of diabetes with caries and periodontitis call for a syndemic approach in adolescence.

## 1. Introduction

Syndemia is the interaction of two or more diseases, co-occurring or sequential, triggered by economic, social, and environmental contextual risks, multiplying the overall burden of diseases in a scenario of social injustice [1,2]. Social vulnerability favors aggregating behavioral risk factors to form a syndemic interplay, like non-communicable disease (NCD) grouping [1].

Syndemic models allow for the investigation of pathways through which inequality contributes to behavioral risk factors resulting in disease grouping. When sifting through the literature, no studies using the syndemic approach to analyze a set of NCDs in adolescence, including oral diseases, were found. Caries and periodontitis are among the most prevalent NCDs worldwide [3]. These oral diseases are economically and socially determined, being more expressed in low- and middle-income countries or deprived populations in high-income settings [4]. Caries and periodontitis share common risk factors with each other and with other NCDs, such as an unhealthy diet high in added sugars [5], smoking, and alcohol consumption [4,5]. 

Caries and periodontitis are mediated by oral biofilm [6]. The excessive intake of fermentable carbohydrates, especially sugars, has been implicated in oral biofilm dysbiosis, resulting in both caries and periodontitis [6]. In addition, an unhealthy diet rich in sugars may contribute systemically to periodontal inflammation resulting from advanced glycation end-products (AGEs), oxidative stress, and inflammation [5,7,8]. Low-grade systemic inflammation has been pointed out as a universal mechanism behind the NCDs [9] and related to caries in childhood and adolescence [5,10]. High sugar intake has been associated with obesity [11], similar to caries and periodontitis, even among young persons [12,13]. 

Caries and gingivitis co-occur in early childhood [14] and seem to predict periodontitis in adult life [15]. Periodontitis, in turn, precedes type 2 diabetes for decades in adults [16]. However, insulin resistance may occur during periodontitis onset in adolescents, suggesting that periodontal diseases and diabetes occur concurrently throughout life [17]. In addition, untreated caries and tooth loss may predict all-cause mortality, especially mortalities due to NCDs, such as cardiovascular and cancer [18].

We have shown the grouping of caries and periodontal indicators, forming the phenomenon of Chronic Oral Disease Burden in young Brazilians [5] and in Americans from adolescence to elderhood [19]. Moreover, we have proposed an Insulin Resistance Phenotype to represent the early events of the diabetes continuum associated with earlier cardiovascular risk events in adolescents [20]. Thus, we hypothesize that a syndemic framework involving socioeconomic inequalities and behavioral risk exposures would result in grouping obesity and the Insulin Resistance Phenotype with caries and periodontitis among adolescents. Therefore, we modeled syndemic pathways from Socioeconomic Inequalities and Behavioral Risk Factors (high sugar consumption, smoking, and alcohol) toward the grouping of obesity and the Insulin Resistance Phenotype with a Chronic Oral Disease Burden at the end of the second decade of life.

## 2. Materials and Methods

### 2.1. Study Design

A population-based study was nested within the Consortium of Brazilian birth cohorts from Ribeirão Preto, Pelotas, and São Luís (RPS Birth cohorts) [21]. This cohort has been studying precursors of noncommunicable diseases at baseline (birth period), and at the end of the first (1st follow-up) and second decades of life (2nd follow-up). 

The birth cohort included 94.1% (*n* = 2541) of all births in São Luís from March 1997 to February 1998 (baseline). From January to November 2016, 687 participants from the initial cohort, aged 18–19 years, were located. At that time, to increase sample power and prevent future losses, the cohort also had an open design (retrospective cohort) that included adolescents born in São Luís in 1997. The retrospective cohort was drawn using the Brazilian Living Birth Information System database (SINASC), generating a random sample (*n* = 1133). Additionally, individuals identified in schools and universities as long as they were registered in the SINASC (*n* = 695) were also included. The final study sample for the present study comprised 2515 adolescents from the original perspective and retrospective cohorts to ensure sample representativeness.

This study was approved by the Ethics and Research Committee of the Federal University of Maranhão University Hospital (IRB #1,302,489). All participants signed informed consent. We reported this study following the STROBE guidelines.

### 2.2. Data Collection

We collected socioeconomic information, including monthly family income, categorized as ≥5, 3 to <5, 1 to <3, or 1 Brazilian national minimum wage in 2016 (USD 252.1); adolescent’s education, categorized as college (incomplete or complete), high school, and middle school; household head’s education, similarly classified; and socioeconomic class using Brazilian Economic Classification from A to E classes, in which Class A is the wealthiest and Class E, the poorest. The adolescent’s sex was recorded as male (1) or female (2).

Smoking was a dichotomous categorical variable defined as current cigarette smoking. Problems related to alcohol use were measured using the Alcohol Use Disorder Identification (AUDIT) [22] and classified as low (score 0 to 4) or high risk (score of 5 or more).

Dietary information was obtained from Food Frequency Questionnaires (FFQ), composed of 106 foods and beverages, including frequency, portion size, and quantity, related to the last 12 months [23]. A quality–quantity FFQ estimated the portion sizes (small, medium, or large) using a photographic record to reduce diet measurement bias. The questionnaire was administered by adequately trained nutritionists using REDCap, a web application for online research and databases. 

Added sugars refer to sugars and syrups incorporated into foods during preparation or processing or added to the table [24]. These sugars are the primary origin of discretionary calories in the human diet and have been implicated with obesity and NCDs, such as diabetes, cardiovascular diseases, caries, and periodontitis [5,14]. The daily added sugars intake (mL or g) was calculated by multiplying the frequency of consumption and the daily recorded portion size from added sugar present in beverages such as soft drinks, fruit-flavored juice, chocolate drinks, energy drinks, and a wide range of food groups, such as dairy products, bread, cookies, breakfast cereals, desserts, chocolate, mayonnaise, salty snacks, and cold cuts. Finally, the added-sugar consumption was estimated as the percentage of calories from sugar of daily total energy intake and the daily sugar intake in grams. The daily sugar limit for adolescents was categorized according to the American Heart Association’s guidelines up to <25 g [24] (ideal), and high exposure as 25 g to 49.9 g, 50 g to 74.9 g, and >75 g per day.

The adolescent’s height (in meters) was elicited using a stadiometer (Altura Exata^®^, Belo Horizonte, MG, Brazil) and the weight (kg) using a dual-energy X-ray absorptiometry (DEXA). The Body Mass Index (BMI) was calculated (kg/m^2^) and used as a categorical variable [25]: <25 kg/m^2^; 25 to <30 kg/m^2^; and ≥30 kg/m^2^.

A blood sample (40 mL) was collected from the cubital vein before a snack was served to the adolescents who were fasting for at least 2 h to analyze the serum level of triglycerides (mg/dL), high-density lipoprotein (HDL) (mg/dL), very low-density lipoprotein (VLDL) (mg/dL), and blood glucose (mg/dL). These markers were measured using the Sysmex XE-2100^®^ (Sysmex Corporation, Kobe, Japan) hematology analyzer [20].

Six dentists examined the caries and periodontal indicators. The training process included 30 h of theoretical and practical aspects. It was performed on 13 adolescents and repeated within 24 h. Oral examinations were conducted under artificial light in a dental unit located within the research facilities. The following clinical parameters were gathered: the number of decayed teeth (DMF-T index) and teeth with visible plaque (VPI) were evaluated by examining four surfaces of all teeth, except for the third molars [26]. Bleeding on probing (BoP) (presence or absence of bleeding after periodontal probing), periodontal probing depth (PPD) (distance from the gingival margin to the most apical extent of probe penetration), and clinical attachment level (CAL) (distance from the cement–enamel junction to the most apical extent of probe penetration) were examined at six sites per tooth, excluding third molars. The inter-examiner Kappa index was 0.82 for the DMFT index, and the Interclass Correlation Coefficient was 0.88 for PPD, 0.84 for BoP, 0.93 VPI, and 0.97 for CAL.

### 2.3. Latent Variables

Latent variables are unobserved variables that reflect complex phenomena of multiple dimensions estimated by the shared variance among their effect indicators (observed variables) [27]. A latent variable estimation is the magnitude of the intercorrelations of their indicators, resulting in an effective estimate free of measurement errors and with greater power to detect differences [27,28]. The latent variables of this study were as follows: *Socioeconomic Inequalities*, *Behavioral Risk Factors*, *Insulin Resistance Phenotype*, and *Chronic Oral Disease Burden*.

Were deduced from the shared variance of the indicators: (a) monthly household income, (b) adolescent’s education, (c) household head’s education, and (d) socioeconomic class. 

*Behavioral Risk Factors* were constructed from the shared variance of the indicators: (a) smoking, (b) alcohol abuse, and (c) added-sugar consumption.

*Insulin Resistance Phenotype* was composed of the shared variance of the indicators: (a) Triglycerides /HDL ratio, (b) VLDL concentration, and (c) TyG index. The TyG index was calculated by multiplying blood glucose by triglycerides, as in the formula Naperian logarithm (Ln [Triglyceride (mg/dL) × fasting glucose (mg/dL)/2]. All these indicators are markers of insulin resistance in young people [20].

*Chronic Oral Disease Burden* was estimated from the shared variance of the indicators: (a) the number of teeth with carious lesions, (b) VPI, (c) the number of teeth with BoP, (d) the number of teeth with PPD ≥4 mm, and (e) the number of teeth with CAL ≥3 mm [4,19,29].

### 2.4. Theoretical Model

**Figure 1:**
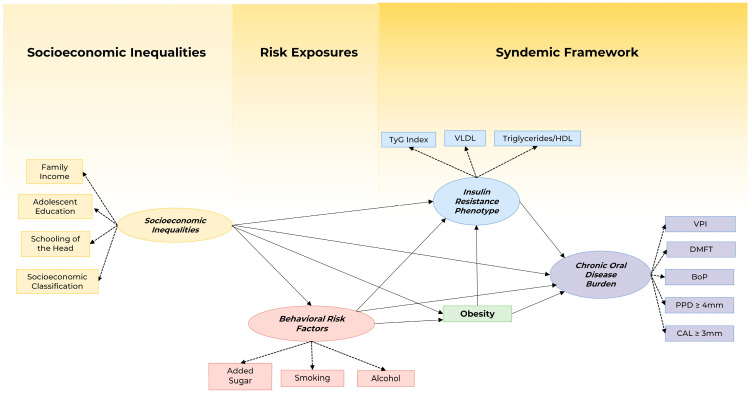
The theoretical model proposed for the analysis of the association between Socioeconomic Inequalities, Behavioral Risk Factors, obesity, Insulin Resistance Phenotype, and Chronic Oral Disease Burden in adolescents.

We constructed a theoretical model to investigate a syndemic framework involving socioeconomic inequalities, behavioral risk factors for NCDs, and the grouping of obesity and early signs of diabetes risk with the co-occurrence of caries and periodontitis in adolescents. *Socioeconomic Inequalities* were the ancestral variable influencing the *Behavioral Risk Factors* resulting in the NCDs outcomes, namely: obesity, *Insulin Resistance Phenotype* with *Chronic Oral Disease Burden*. The model was adjusted for sex (Figure 1).

### 2.5. Statistical Analysis

Structural equation modeling (SEM) is an epidemiological tool allowing for the construction of latent variables and the interpretation of the results of multiple regressions simultaneously, assisting in evaluating variables involved in complex phenomena and minimizing biases arising from measurement errors [27].

The latent variables of *Socioeconomic Inequalities*, *Behavioral Risk Factors*, *Insulin Resistance Phenotype*, and *Chronic Oral Disease Burden* were constructed based on exploratory factor analysis and confirmatory factor analysis [27].

As a sensitivity analysis, due to the high correlation between smoking and alcohol consumption, we also analyzed a model with the interaction between these two variables by multiplying their indicators.

The weighted least squares estimator with mean and variance fit (WLSMV) and Theta parameterization were performed to control for residual variance. The evaluation of the overall fit quality was assessed using the indicators: (a) Root Mean Square Error of Approximation (RMSEA) with the upper bound of the 90% confidence interval below 0.08 and (b) Comparative Fit Index (CFI) and Tucker–Lewis Index (TLI) > 0.90 [27]. We assumed the standardized coefficient (SC) as significant if *p* < 0.05. Missing data were imputed by Maximum Likelihood Estimation (MLE), assuming that these data were missing randomly [20]. We performed the analyses using the Mplus^®^ 8.0 software.

## 3. Results

Among the 2515 adolescents enrolled in the study, 3.56% (*n* = 89) were smokers, 19.43% (*n* = 488) had a higher risk for alcohol abuse, 6% (*n* = 151) had BMI ≥30 kg/m^2^, and 81.35% (*n* = 2033) consumed >25 g of added sugar per day. Regarding insulin resistance, 13.76% (*n* = 346) had TG/HDL-C ratio ≥3 mg/dL, 16.98% (*n* = 427) had VLDL-c above 30 mg/dL, and the mean TyG index was 8.22 mg/dL. The mean number of decayed teeth was 1.17 (SD ± 1.2). The mean number of teeth with PPD ≥4 mm was 1.17 (±2.47), with CAL ≥3 mm was 13.98 (±7.20), and with BoP was 11.65 (±6.67) (Table 1).

The fit indices indicated a good fit for the proposed model: RMSEA (0.067), 90% CI (0.064–0.070), CFI (0.938), and TLI (0.920) (Table 2). All effect indicators for the latent variables *Socioeconomic Inequalities*, *Insulin Resistance Phenotype*, and *Chronic Oral Disease Burden* showed convergent factor loadings (SC > 0.3; *p* < 0.001) (Table 3).

*Socioeconomic Inequalities* were associated with a higher *Chronic Oral Disease Burden* in adolescents (SC = 0.222; *p* < 0.001). Meanwhile, *Socioeconomic Inequalities* were inversely associated with obesity (SC = −0.099; *p* = 0.010) (Table 4).

There was a strong correlation between the indicators of the latent variable *Behavioral Risk Factors* (*p* < 0.001) (Table 3), which was associated with *Chronic Oral Disease Burden* (SC = 0.102; *p* = 0.013). 

Proximally, revealing a syndemic framework, we observed the grouping of NCDs. Obesity was associated with the *Insulin Resistance Phenotype* (SC = 0.098; *p* < 0.001) and the *Chronic Oral Disease Burden* (SC = 0.052; *p* = 0.033). The *Insulin Resistance Phenotype* and the *Chronic Oral Disease Burden* were also associated (SC = 0.089; *p* = 0.007) (Table 4).

Girls had lower levels of *Behavioral Risk Factors* (SC = −0.216; *p* < 0.001) and *Chronic Oral Disease Burden* (SC = −0.141; *p* < 0.001).

Supplementary Table S2 shows a significant correlation between the insulin resistance indicators (Triglycerides /HDL, TyG index, and VLDL) with each other, with obesity, and with the periodontal disease indicators. Caries and periodontal disease indicators were associated with each other. 

## 4. Discussion

We highlighted a syndemic framework linking obesity and the *Insulin Resistance Phenotype* with the *Chronic Oral Disease Burden* at the end of the second decade of life. *Socioeconomic Inequalities* were associated with a higher *Chronic Oral Disease Burden* in adolescents. *Behavioral Risk Factors* were associated with *Chronic Oral Disease Burden*. 

Although NCDs’ co-occurrence has been previously shown, studies frequently analyzed the association of two conditions only, such as caries and periodontitis [4]; obesity and caries [13]; obesity and periodontitis [12]; obesity and insulin resistance [30]; and insulin resistance and periodontitis [10]. Our findings are pioneers in identifying the co-existence of multiple conditions by the end of the second decade of life, namely, insulin resistance, obesity, caries, and periodontitis. These findings stimulate a reflection on the approaches toward oral disease prevention and treatment, based mainly on intervention models targeting the oral biofilm [31]. Moreover, it supports more effective recommendations for tackling NCDs in youth, targeting socioeconomic, commercial determinants, and behavioral risk factors. This would impact not only the oral disease burden but also reduce rates of obesity, diabetes, and other NCDs in the future. 

In epidemiological studies, we have used the *Chronic Oral Disease Burden* as a latent variable to analyze the correlation between caries and periodontitis indicators [4,19,29]. We draw attention to the fact that the *Chronic Oral Disease Burden* is not a diagnostic tool to be used in the clinical setting; instead, it is an epidemiological approach for understanding why the indicators of caries and periodontitis group through life and investigating their common risk factors [4,19,29]. In addition, this latent variable allows us to analyze the periodontal indicators in a continuous manner, dispensing cut-off points to determine case definition, which persists in disagreement and remains challenging in younger populations [32,33].

Intermediately, higher exposure to *Behavioral Risk Factors* increased the *Chronic Oral Disease Burden*. Strategies encompassing economic, social, structural, and commercial determinants for these behavioral risks may be more effective in reducing the burden of NCDs, including oral ones [34]. We cite, as examples of public policy measures, the successful implementation of anti-smoking laws in countries like Brazil [35], the regulation of access to alcoholic beverages, and market regulations that include the taxation, labeling, and regulation of sugar contents in products [36]. As alarming, we identified a set of *Behavioral Risk Factors* adopted by adolescents—sugar consumption, smoking, and alcohol abuse—that converged toward the latent variable *Behavioral Risk Factors*. While these variables measured different conditions, their convergence might be understood. For instance, sugar activates the central nervous system’s hedonic reward mechanism, inducing a dependence similar to addictive drugs [37], explaining its correlation with alcohol and smoking.

Ancestrally, as the primary determinant of health in our syndemic model, *Socioeconomic Inequalities* increased the *Chronic Oral Disease Burden*, reflecting aspects related to low education, deprived access to health services, insufficient oral hygiene practices and self-care, and food insecurity [38]. The social determinants of health are a universal phenomenon identified in low-, middle- and high-income countries, where socioeconomic disparities determine the poorest oral health indicators [39]. Oral diseases mainly affect disadvantaged and socially marginalized populations, showing that people experience health inequalities according to their position on the social scale [39].

Unexpectedly, in this study, higher *Socioeconomic Inequalities* were inversely associated with obesity. Brazil is currently experiencing a nutritional transition, shifting from nutritional deficit to obesity, especially among the poorest. In this context, in São Luís, the state capital with the lowest Human Development Index in Brazil, only 4.1% of the adolescents were obese, whereas, in more affluent regions of the country, the prevalence of obesity ranges from 6.6% to 11.1% [40]. Thus, it becomes evident that patterns of socioeconomic inequalities associate differently with obesity across the different Brazilian regions.

The sensitivity analysis revealed that high sugar consumption and the interaction between smoking and alcohol consumption were associated with the *Chronic Oral Disease Burden* (Table S1). The role of sugars in the etiology of caries is well-known, where the metabolism of sugars by dental biofilm results in dysbiosis, pH drop, and, consequently, tooth demineralization [6]. Concerning periodontitis, sugar may act locally, resulting in biofilm accumulation and dysbiosis [6,8], and systemically, involving oxidative stress and low-grade systemic inflammation [8,41]. Our findings shed light on the high number of Brazilian adolescents consuming sugar at a rate of above 25 g/day, considered the highest cutoff point for future cardiovascular diseases, according to the American Heart Association [24]. Smoking consumption is recognized as a cause of periodontitis [42]. As our population was composed of adolescents, it is relevant to consider that the harmful effects of smoking and alcohol are dose-dependent and cumulative [43].

In the sensitivity analysis, we observed that *Socioeconomic Inequalities* increased added-sugar consumption by adolescents. Inequalities lead to an unsafe environment, favoring unhealthy risk behaviors and resulting in exposure to processed foods rich in sugar, besides smoking and alcohol [36]. Low-income populations are more exposed to unhealthy diets, sugar-rich foods, and beverages that are cheaper and more accessible to their purchasing power [44].

Limitations of our study include its cross-sectional design, which prevents the drawing of causal relationships between the presumed exposures and outcomes. However, without the pretension to assume temporality, we highlighted a syndemic framework that requires common strategies to tackle multiple NCDs simultaneously. Fasting for 2 h (minimum) instead of extended periods (8 to 12 h) could be pointed out as a study limitation when evaluating insulin resistance. However, fasting has little effect on lipid profile measurements, resulting in international guidelines stating that blood analysis can be performed without fasting [45,46,47]. Furthermore, the Insulin Resistance Phenotype was analyzed as a continuous variable dispensing cutoff value, which represented the shared variance values among triglycerides/HDL, TyG, and VLDL, reducing measurement errors for any isolated indicator [20]. 

As strengths of our study, we showed the occurrence of a grouping of NCDs in adolescence: obesity, diabetes precursors, caries, and periodontitis. Moreover, the SEM analytical approach allowed for the studying of multiple outcomes, including complex conditions analyzed as latent variables, such as the *Insulin Resistance Phenotype* and the *Chronic Oral Disease Burden*, reducing the measurement error of these phenomena. 

Adolescence is one of the most sensitive periods of human development, representing a “window of opportunity” for health interventions since several behaviors that begin at this life stage may affect future health [48]. We identified the co-occurrence of obesity and the early events of diabetes with caries and periodontitis at the end of the second decade of life. Our findings alert the need for a syndemic approach to adolescent health, directing efforts toward social, economic, and commercial determinants and behavioral risk factors to address NCDs, including oral diseases.

## Figures and Tables

**Table 1:** Sociodemographic, behavioral risk, oral disease, and metabolic risk indicators in adolescents (São Luís, Brazil, 2016).

Variables	*N*	%	Mean	Standard Deviation
Income, Brazilian minimum monthly wage				
≥5	285	11.33	-	-
3 to <5	338	13.44	-	-
1 to <3	1079	42.90	-	-
<1	797	31.69	-	-
Missing	16	0.64		
Educational level of the adolescent				
College (incomplete)	672	26.72	-	-
High school	1758	69.90	-	-
Middle school	83	3.30	-	-
Missing	2	0.08	-	-
Educational level of the head of the family				
College	325	12.92	-	-
Incomplete college	81	3.22	-	-
High school	1260	50.10	-	-
Middle school	563	22.39	-	-
Illiterate	286	11.37	-	-
Family economic class (ABEP)				
A	94	4.22	-	-
B	565	25.37	-	-
C and D	1118	50.20	-	-
E	450	20.21		
Sex				
Male	1197	47.59	-	-
Female	1318	52.41	-	-
Adolescent age				
18 years	1742	69.26	-	-
19 years	773	30.74	-	-
Added sugar				
<25 g	466	18.65	-	-
25 g a 49.9 g	705	28.21	-	-
50 g a 74.9 g	526	21.05	-	-
>75 g	802	32.09	-	-
Smoking				
Yes	89	3.56	-	-
No	2414	96.44	-	-
Risk of alcohol abuse				
High risk	488	19.43	-	-
Low risk	2.023	80.57	-	-
Height (cm)	166.81	9.11	-	-
Weight (kg)	61.48	13.13	-	-
Body Mass Index (BMI)				
Non-obese (BMI < 25 kg/m^2^)	1905	75.75	-	-
Overweight (BMI ≥ 25 to <30 kg/m^2^)	459	18.25	-	-
Obese (BMI ≥ 30 kg/m^2^)	151	6.00	-	-
Triglycerides	-	-	91.01	49.28
HDL	-	-	49.37	11.92
Blood glucose level	-	-	91.98	15.78
Triglycerides /HDL	-	-	2.05	1.72
VLDL	-	-	18.18	9.73
TyG	-	-	8.22	0.46
Number of decayed teeth	-	-	1.58	2.14
Visible plaque index (%)				
<15%	952	39.97	-	-
≥15%	1430	60.03	-	-
Bleeding on probing	-	-	11.65	6.67
Periodontal probing depth ≥ 4	-	-	1.17	2.47
Clinical attachment level ≥ 3 mm	-	-	13.98	7.20

**Table 2:** Adjustment measures of the structural equation model to analyze the association between *Socioeconomic Inequalities*, *Behavioral Risk Factors*, obesity, *Insulin Resistance Phenotype*, and *Chronic Oral Disease Burden* in adolescents (São Luís, Brazil, 2016).

Estimators	Expected Indices	Model Indices
*X*^2^ *		475.064
Degrees of freedom		120
*p* value *X*^2^		0.0000
RMSEA ^†^	<0.05	0.067
90% CI ^‡^	<0.08	0.64–0.070
*p* ^§^	>0.05	0.890
CFI ^||^	>0.90	0.938
TLI ^#^	>0.90	0.920

* Chi-squared test. ^†^ Root means square error of approximation. ^‡^ Confidence interval. ^§^ *p* value. ^||^ Comparative fit index. ^#^ Tucker–Lewis index.

**Table 3:** Factor loading, standard error, and *p*-value for the effect indicators of the latent variables: *Socioeconomic Inequalities*, *Behavioral Risk Factors*, *Insulin Resistance Phenotype*, and *Chronic Oral Disease Burden* (São Luís, Brazil, 2016).

Latent Variable	Standardized Coefficient	Standardized Error	*p*
*Socioeconomic Inequalities*			
Household income	0.621	0.026	<0.001
Educational level of the adolescent	0.529	0.023	<0.001
Educational level of the head of the family	0.712	0.024	<0.001
Economic class	0.854	0.027	<0.001
*Behavioral Risk Factors*			
Added Sugar	0.221	0.038	<0.001
Smoking	0.995	0.098	<0.001
Alcohol abuse	0.628	0.066	<0.001
*Insulin Resistance Phenotype*			
Triglycerides/HDL	0.770	0.052	<0.001
VLDL	0.902	0.061	<0.001
TyG Index	0.939	0.063	<0.001
*Chronic Oral Disease Burden*			
Decayed component (DMFT Index)	0.335	0.018	<0.001
Visible Plaque Index (VPI Index)	0.605	0.015	<0.001
Bleeding on probing	0.516	0.018	<0.001
Periodontal probing depth ≥ 4 mm	0.674	0.013	<0.001
Clinical attachment level ≥ 3 mm	0.672	0.013	<0.001

**Table 4:** Standardized coefficient, standard error, and *p* value for the total effects of the association between Socioeconomic Inequalities, Insulin Resistance Phenotype, Chronic Oral Diseases Burden, added sugar, and smoking and alcohol interaction in adolescents (São Luís, Brazil 2016).

Explanatory Variables	Outcome	Standardized Coefficient	Standardized Error	*p*
*Socioeconomic Inequalities*	*Chronic Oral Diseases Burden*	0.222	0.026	<0.001
*Socioeconomic Inequalities*	Obesity	−0.099	0.032	0.010
Obesity	*Insulin Resistance Phenotype*	0.098	0.027	<0.001
Obesity	*Chronic Oral Diseases Burden*	0.089	0.033	0.007
*Insulin Resistance Phenotype*	*Chronic Oral Diseases Burden*	0.052	0.025	0.033
*Behavioral Risk Factors*	*Chronic Oral Diseases Burden*	0.102	0.041	0.013
Sex	*Chronic Oral Diseases Burden*	−0.141	0.024	<0.001
Sex	*Behavioral Risk Factors*	−0.216	0.036	<0.001

## Data Availability

The data that support the findings of this study are available on request from the corresponding author. The data are not publicly available due to privacy or ethical restrictions.

## Supplementary Materials

The following supporting information can be downloaded at: https://www.mdpi.com/article/10.3390/nu15163512/s1, Supplementary Table S1. Sensitivity analysis including the standardized coefficient, standard error, and *p* value for the total effects of the association between Socioeconomic Inequalities, Insulin Resistance Phenotype, Chronic Oral Diseases Burden, added sugar, and smoking and alcohol interaction in adolescents (São Luís, Brazil, 2016). Supplementary Table S2. Correlation matrix between the indicators of Insulin Resistance Phenotype, obesity, and Chronic Oral Disease Burden (São Luís, Brazil, 2016).
