# Supplementary material for: Obesity, Insulin Resistance, Caries, and Periodontitis: Syndemic Framework
Source: Nutrients. 2023 Aug 9;15(16):3512. doi: 10.3390/nu15163512 (PMC10458482; doi:10.3390/nu15163512)
Supplement: Supplementary file 1 [file nutrients-15-03512-s001.zip › nutrients-2450165-SI.pdf]

Supplementary Table S2. Correlation matrix between the indicators that form the outcomes Insulin Resistance Phenotype, Obesity and Chronic Oral Disease Burden (São Luís, Brazil, 2016).

	**TyG**	**TG/HDL**	**VLDL**	**Obesity**	**Carie**	**BoP**	**PPD ≥ 4mm**	**CAL ≥ 3mm**
**TyG**	1.0000							
**TG/HDL**	0.7219<br>P<0.001	1.0000						
**VLDL**	0.8469<br>P<0.001	0.8350<br>P<0.001	1.0000					
**Obesity**	0.0674<br>P<0.001	0.0425<br>P=0.042	0.0660<br>P<0.001	1.0000				
**Carie**	0.0195<br>P=0.362	0.0131<br>P=0.538	0.0113<br>P=0.597	0.0207<br>P=0.314	1.0000			
**BoP**	0.0394<br>P=0.065	0.0190<br>P=0.375	0.0248<br>P=0.246	0.0286<br>P=0.165	0.0930<br>P<0.001	1.0000		
**PPD ≥ 4**	0.0442<br>P=0.039	0.0398<br>P=0.063	0.0419<br>P=0.050	0.0344<br>P=0.094	0.2110<br>P<0.001	0.3590<br>P<0.001	1.0000	
**CAL ≥ 3mm**	0.0667<br>P<0.001	0.0566<br>P<0.001	0.0622<br>P<0.001	0.0545<br>P<0.001	0.1826<br>P<0.001	0.2985<br>P<0.001	0.4887<br>P<0.001	1.0000

TG/HDL - Triglycerides /HDL ratio BoP - Number of teeth with bleeding on probing; PPD≥4mm - Number of teeth with probing depth ≥4mm; CAL≥3mm - Number of teeth with clinical attachment level ≥3mm.

Supplementary Table S1. Sensitivity analysis including the standardized coefficient, standard error and p value for the total effects of the association between Socioeconomic Inequalities, Insulin Resistance Phenotype, Chronic Oral Diseases Burden, added sugar and smoking and alcohol interaction in adolescents (São Luís, Brazil, 2016).

Explanatory variables	Outcome	Standardized coefficient	Standardized error	*p*
Socioeconomic Inequalities	*Chronic Oral Diseases Burden*	0.210	0.027	<0.001
Socioeconomic Inequalities	Obesity	-0.056	0.023	0.015
Socioeconomic Inequalities	Added sugar	0.102	0.026	<0.001
Added sugar	Obesity	-0.104	0.024	<0.001
Added sugar	*Chronic Oral Diseases Burden*	0.094	0.029	<0.001
Obesity	*Chronic Oral Diseases Burden*	0.069	0.023	0.003
Obesity	*Insulin Resistance Phenotype*	0.072	0.019	<0.001
Insulin Resistance Phenotype	*Chronic Oral Diseases Burden*	0.053	0.025	0.032
Smoking and Alcohol Interaction	*Chronic Oral Diseases Burden*	0.115	0.055	0.036
Smoking and Alcohol Interaction	Added sugar	0.189	0.055	0.001
Sex	*Chronic Oral Diseases Burden*	-0.145	0.024	<0.001
Sex	Obesity	0.057	0.021	0.008
Sex	Smoking and Alcohol Interaction	-0.163	0.052	0.002
